# Aqueous ethanol extract of *Libidibia ferrea* (Mart. Ex Tul) L.P. Queiroz (juca) exhibits antioxidant and migration-inhibiting activity in human gastric adenocarcinoma (ACP02) cells

**DOI:** 10.1371/journal.pone.0226979

**Published:** 2020-01-17

**Authors:** Luana França Calandrini de Azevedo, Tássia Alana Alves Ferreira, Karina Motta Melo, Clara Louise Porfírio Dias, Carlos Eduardo Matos Carvalho Bastos, Seidel Ferreira Santos, Alberdan da Silva Santos, Cleusa Yoshiko Nagamachi, Julio Cesar Pieczarka

**Affiliations:** 1 Laboratório de Citogenética, Centro de Estudos Avançados da Biodiversidade, Instituto de Ciências Biológicas, Universidade Federal do Pará, Belém, Pará, Brazil; 2 Laboratório de Investigação Sistemática em Biotecnologia e Biodiversidade Molecular, Universidade Federal do Pará, Belém, Pará, Brazil; University of British Columbia, CANADA

## Abstract

*Libidibia ferrea* (juca) is a plant belonging to the Fabaceae (Leguminosae) family, whose antioxidant activity has been widely described in the literature. We evaluated this parameter of Aqueous ethanol extract (AE), ethyl acetate (ACO), chloroform (CLO) and hexane (HEX) extracts of *L*. *ferrea*. We then tested the most active extract for its toxicity and ability to inhibit migratory activity in the ACP02 gastric adenocarcinoma cell line *in vitro*. The AE and ACO extracts both had antioxidant activity, the AE extract showing greater potential. This may reflect that both extracts contained phenolic compounds. Although AE extract showed no cytotoxic, mutagenic or genotoxic effect, it altered cell morphology and migration activity. Analysis of apoptosis/necrosis indicated that this parameter does not appear to account for the apparent ability of AE to inhibit cancer cell migration. We speculate that the morphological changes in AE-treated cells could be due to cytoskeleton alterations related to the presence of myo-inositol in AE extract. Together, our results demonstrate this extract of *L*. *ferrea* can act as an exogenous antioxidant and might prove useful in efforts to fight secondary tumors.

## 1. Introduction

The multiple stages of cancer range from alteration of genetic material to the migration and establishment of altered cells in neighboring tissues and organs. This process of dissemination, known as metastasis, leads to aggravation of the disease and hinders conventional treatment [1]. For this reason, it is important that we identify additional safe and efficient antimetastatic substances.

The accumulation of empirical knowledge regarding medicinal plants has facilitated their increasing use as complementary and alternative therapies for numerous diseases, including cancer [2]. *Libidibia ferrea*, which is commonly called juca or pau-ferro, belonging to the Fabaceae (Leguminosae) family. The *L*. *ferrea* is an Angiosperm medium-size tree previously classified as *Caesalpinia ferrea*, Caesalpininoideae subfamily and Caesalpinieae tribe [3]. This is a monophyletic family with the following synapomorphies: leaves composite, alternate, with pulvinus; petal differentiated adaxial and monocarpellary ovary [4]. The fruits are usually from the legumen type with some variations [5]. The plants are presented as annual herbs or perennial, erect, prostrate, diffuse, vines, lianas, undergrowth, shrubs and small, medium or large trees [6]. That has been widely utilized in traditional medicine as an antipyretic, antidiabetic and wound-healing agent. Research has shown that this plant has anti-inflammatory, analgesic, anticoagulant, antiulcerogenic, antihistaminic and cancer-preventing activities [7,8]. Furthermore, it has been widely cited in the literature for its antioxidant activity [9,10].

Studies have correlated the antioxidant potential of *L*. *ferrea* extracts with the presence of phenols [11,12], which exert antioxidant activities by preventing or retarding oxidation via the blockade/capture of free radicals [13]. It has been proposed that juca can act as an exogenous antioxidant by preventing free radicals from interacting with fundamental molecules of the organism to cause cellular instability and trigger pathologies such as cancer [14]. Thus, the juca became a plant of interest because it is widely used by the population based on empirical knowledge, but without studies related to its activity in cancer cells, including its action in preventing new tumor formation and cell migration.

*In vitro* assays can be used to examine the safety of plant preparations and their phytochemical constituents [15], while wound-healing assays and other tests can be used to evaluate the ability of plant preparations to inhibit aspects of tumorigenesis. Indeed, many medicinal plants and their constituents have been shown to inhibit the migratory capacity of cancer cells [16,17]. The ACP02 cell line is often used for this type of research [18,19] because it shares important traits with its tumor of origin, including amplification of the *MYC* oncogene and deletion of the *TP53* tumor suppressor gene. As most cancer are characterized by a high degree of metabolic activity, ACP02 cells displays the requirements to be used in the research and a good model for the *in vitro* screening of anticancer drugs [20,21].

Here, we obtained four extracts from the pods of juca and assessed them for antioxidant activity. The most active extract was tested for its toxicity and inhibition of cell migration in ACP02 cell line.

## 2. Materials and methods

### 2.1 Collection of samples

The pods of *L*. *ferrea* were collected in the city of Marabá/PA (latitude 05°22’07”S, longitude 49°07’04”W), in July 2014 (authorization number 13248). The plant was identified, by botanist Seidel Santos and a voucher sample (n^o^002780) was deposited in the MFS herbarium of the Universidade do Estado do Pará (UEPA). JCP has a permanent field permit, number 13248 from “Instituto Chico Mendes de Conservação da Biodiversidade”. The Cytogenetics Laboratory from UFPa has permit number 19/2003 from the Ministry of Environment for sample transport and permit 52/2003 for using the samples for research. The Ethics Committee (Comitê de Ética Animal da Universidade Federal do Pará) approved this research (Permit 68/2015).

### 2.2 Preparation of extracts

Dried and powdered pods (300 g) were subjected to selective extractions with organic solvents in the following order of polarity: n-hexane, chloroform, ethyl acetate and alcohol 70% solution. The solvent: material ratio was 2:1 and the mixture was subjected to the extraction. Ultrasound-assisted extraction was performed in an ultrasonic cleaner bath (USC-1800) with a volume of 9 L, an input power of 155 W, 40 KHz of frequency, and at 30°C (± 3) and 30 min for hexane (HEX), chloroform (CLO) and acetate (ACO) extracts; and 45°C (± 3) and 30 min for aqueous ethanol extract (AE). The ultrasonic power inside de extract container was estimated to 70 W.cm-2. The extracts were concentrated with a Buchi R3 rotary evaporator (V 700 vacuum pump, V 850 vacuum controller) was used to remove the solvent at 45°C and 156 mbar, 207 mbar, 240 mbar, 240 mbar and 58 mbar pressure, respectively [22].

### 2.3 Chemical characterization of samples

#### 2.3.1 Derivatization

Derivatization was performed as described by [23]. For the dried HA, ACO and CLO extracts, 5 mg of extract was resuspended in 100 μL of the derivatization reagent, N,O-bis(trimethylsilyl)-trifluoroacetamide (BSTFA), with stirring at 600 rpm for 15 min at 45°C. For the HEX extract, 5 mg of dried extract was resuspended in NaOH+MeOH (9:1) at 45°C for 20 min, 500 μL of hexane:ether (1:1) was added, and the mixture was stirred (45°C/5 psi/60 min). The solution was evaporated to dryness, and the lipid residue was resuspended in 100 mL of BSTFA with stirring at 600 rpm/45°C for 15 min.

#### 2.3.2 Metabolite identification (GC/MS)

Gas chromatography/mass spectrometry (GC/MS) was performed following the description of [24]. We used a Thermo Scientific Trace 1300 GC device coupled to a Thermo Scientific MS-ISQ Single Quadrupole mass spectrometer with an AI 1310 autosampler, which was equipped with a RTX-65 TG column (15 m x 0.25 mm x 0.1 μm), a DB-5 column (15 m x 0.25 mm x 0.1 μm) or a similar column. Helium gas was used as the carrier at a flow rate of 1 mL/min. The sample (1.0 μL) was injected in the Split mode at a ratio of 1:5. The injector was operated at 250°C. The oven temperature began at 40°C and then ramped up to 200°C at 6°C/min, remained there for 1 min, increased to 300°C at 15°C/min, remained there for 5 min, increased again to 340°C at 15°C/min, and remained there for 9 min. The MS-ISQ parameters were set as follows: interface, 280°C; ionization source, 280°C; mass range, 40–1000 Da; and electronic ionization, 70 eV. The substances were identified by comparing their mass spectra with those listed in commercial libraries NIST2011-WILEI2009-FAMES2011. The triterpene concentration was determined by calculating the normalized peak area.

### 2.4 DPPH (2,2-difenil-1-picrilhidazil) test

The antioxidant activities of the four juca extracts were assayed in triplicate using the procedure described by [25] with some modifications. Briefly, DPPH was diluted to 0.04 mg/mL in methanol, and 900 μL of DPPH solution was mixed with 100 μL of extract diluted in methanol to a concentration of 6.25 μg/mL or 400 μg/mL. Each mixture was kept for 20 min in a light-protected place. The absorbance (OD) was measured at 515 nm using a spectrophotometer (Epoch, Biotek) and the Gen5 (2003) version 2.03.01 software. The results were expressed as the percentage of DPPH radical inhibition (%Inhibition) using the following equation: %Inhibition = {[A1-{(A2+A3)]/A1]/100}, where A1 is the DPPH absorbance, A2 is the DPPH absorbance + extract, and A3 is the methanol absorbance + extract. We then calculated the concentration of extract required to capture 50% of the free radical DPPH (EC50), using linear regression analysis performed with the aid of the Graphpad Prism software, version 6.01.

### 2.5 ABTS [2,2’-azino-bis(3-ethylbenzothiazoline)- 6-sulfonic acid] test

The ABTS antioxidant test was performed according to literature [26] with modifications. ABTS was dissolved in water at a concentration of 7 μM and mixed with Potassium Persulphate at a concentration of 2.45 μM, in the absence of light, at room temperature 12 to 16 h before use for use of radical cation (ABTS +). For the test, an ABTS + solution diluted in water was prepared to an absorbance of 0.700 ± 0.02 at 734 nm. Once the allowed absorbance was reached, 3 ml of the solution was mixed at 100 μl of different filters from each extracted test. After 6 min of reaction the absorbance was read on the spectrophotometer. To calculate the percentage inhibition of the ABTS radical at each concentration, the following equation was used: % inhibition = [(A blank—sample) / A blank] x 100 where "A" is an absorbance of each well and "blank" or one well without medium or cells. The EC50 was estimated as a useful % inhibition at each concentration.

### 2.6 ORAC (Oxygen Radical Absorbance Capacity) test

The ORAC protocol was adapted from the method previously developed [27] and then modified [28,29] for microplates using fluorescein. The analysis was performed on 96-well fluorimetry microplates (Greiner-Germany) and on a Microplate Fluorescence Reader fluorimeter—Bio-Tek Instruments, Inc (USA). A 25 μL volume of the sample was mixed with 150 μL fluorescein (55.5 nM) and incubated for 15 min at 37°C in the microplate before automatically injecting 25 μL AAPH solution (153 mM). The fluorescence was followed for 50 min by readings (λexcitation = 485 nm; λemission = 520 nm). Trolox solutions were prepared for the calibration curve. All solutions were diluted in phosphate buffer (75 mM, pH 7.4).

### 2.7 Cell line, culture conditions and biological assays

ACP02 gastric adenocarcinoma cell line was kindly provided by the Laboratory of Human Cytogenetics and Toxicological Genetics (UFPA). The cells were cultured in RPMI supplemented with 10% fetal bovine serum (FBS), amphotericin (2.5 μg/mL), penicillin (100 IU/mL), streptomycin (100 μg/mL), tylosin (8 μg/mL), ciprofloxacin (10 μg/mL), L-glutamine (0.1 mg/L), and sodium bicarbonate (2.2 mg/L). The flasks were maintained in a 5% CO_2_ incubator at 37°C.

#### 2.7.1 Evaluation of cytotoxicity

Cytotoxicity was evaluated using the MTT test (3-(4,5-dimethylthiazol-2-yl)-2,5-diphenyltetrazolium bromide) following the protocol described by [30] with alterations. A total of 5x10^4^ cells were seeded to each well of a 96-well plate, which was incubated in a CO_2_ incubator at 37°C. After 24 h, triplicate samples were tested with seven concentrations of HA extract: 6.25 μg/mL, 12.5 μg/mL, 25 μg/mL, 50 μg/mL, 100 μg/mL, 200 μg/mL and 400 μg/mL. As a negative control (NC), the cells were exposed to the RPMI culture medium. As a positive control (PC) we used doxorubicin at the concentration of 200 μg/mL. As a vehicle control, cells were exposed to 0.1% DMSO in medium. After 24 or 48 h, the cells were exposed to 100 μL of MTT (5 mg/mL) for 3 h, MTT was removed and 100 μl of DMSO was added to each well. After 1 h, the absorbance at 570 nm was measured using a spectrophotometer (Epoch Biotek) and the Gen5 (2003) software, version 2.03.1. Cell viability (%S) was determined from the formula: %S = 100x[(Atested–Ablank)/ (Anegativecontrol-Ablank)], where “A” is the absorbance of each well and “blank” refers to a well without medium or cells.

#### 2.7.2 Evaluation of the genotoxicity and mutagenicity of Jucá HA extract

We evaluated the DNA-level effects of juca AE extract at concentrations of 25 μg/mL, 50 μg/mL, 100 μg/mL and 200 μg/mL, according to the scheme proposed [31]. As a positive control for the micronucleus test, we used colchicine (0.02 μg/mL), as suggested [32]. As a positive control for the comet test, we used H_2_O_2_ (100 μM), as proposed [33]. As a negative control, we used RPMI alone. As a vehicle control, we used 0.1% DMSO diluted in medium.

#### 2.7.3 Mutagenicity test: Micronucleus assay

The micronucleus test was performed according to the standards listed in [24]. Cells were seeded to a six-well plate at 2.02 x 10^5^ cells/well, cytochalasin B was added to 6 μg/mL, and the plate was incubated for 24 h. After 24 h of exposure, the cells were trypsinized, transferred to a Falcon tube, and mixed with 5 mL of hypotonic solution (KCl). After 3 min, 2 mL of Carnoy fixative with methanol and acetic acid (3:1) was added. The mixture was centrifuged, the supernatant was mixed with 1 mL of Carnoy fixative, and the tube was stored. This analysis was performed in a blinded manner, and 1000 cells per sample were analyzed. The cytokinesis-block proliferation index (CBPI) was generated using the formula CBPI = [M1+2(M2)+3(M3)+4(M4)]/N, where M1 to M4 represent the numbers of cells with 1, 2, 3 and 4 nuclei, respectively, and N is the total number of viable cells. 500 total cells were counted and the binucleate cell count remained until 1000 cells were counted for micronucleus observation and for determination of its frequency through the formula fMN = n°MN/1000 [34].

#### 2.7.4 Genotoxicity test: Comet assay

The comet assay was performed according to the methodology proposed by [35], with adaptations. A total of 5x10^5^ cells were seeded in 25 cm^2^ bottles and incubated in CO_2_ incubators for 24 h. The cells were then exposed to the juca AE extract for 3 h, trypsinized and mixed with 0.5% low-melting-point agarose (20 μL cell suspension in agarose). The mixture was placed on slides pre-coated with normal 1.5% agarose, and coverslips were used to cover the samples. The slides were incubated for 15 min at 4°C and then exposed to lysis solution (2.5 M NaCl, 100 mM EDTA and 10 mM Tris, pH 10.0–10.5) containing 1% Triton X-100 and 10% DMSO. After 24 h, the slides were transferred to a horizontal electrophoresis cube, covered with alkaline buffer (300 mM NaOH and 1 mM EDTA, pH>13) for 30 min, and subjected to electrophoresis for 30 min at 0.8 V/cm. The slides were neutralized by three 5-min washes with deionized water, fixed for 5 min in ethylic alcohol P.A., dried, and stored in a refrigerator until they were subjected to ethidium bromide staining and analyzed under fluorescence microscopy (Nikon H550S with a 510 to 560-nm filter, a 590-nm filter barrier and a zoom of 400x). Comets were categorized based on their tail sizes [36] and 300 cells were analyzed from each group. The damage index (DI) was calculated using the formula: DI = [(1×n1)+(2×n2)+(3×n3)+(4×n4)/n]x100, where n is the total number of analyzed cells and n1 to n4 indicate the numbers of cells with damage levels from 0 to 4.

### 2.8 Cell migration assay

Cell migration was tested using a modification of the previously described wound-healing assay [37]. Cells were seeded to six-well plates at 2.02 x10^5^ cells/well and incubated for 24 h to allow monolayers to form, and a slit was made in each monolayer. The wounded monolayers were exposed to the AE extract at concentrations of 25 μg/mL, 50 μg/mL, 100 μg/mL and 200 μg/mL. RPMI alone was used as the negative control and 0.1% DMSO in medium was used as the vehicle control. The plates were placed in a CO_2_ incubator and photographed after 12, 24 and 48 h, and cellular migration was analyzed using the ToupView software, version 3.5. For our statistical analysis, the wounded areas were measured using the ImageJ software. To calculate the slit-opening (SO) percentage, the values measured at the time of wounding (T0) were taken as 100%, while the other percentages were calculated using the formula %SO = CTx * 100%/ CT0, where C represents the length in pixels and Tx is the analyzed time.

### 2.9 Apoptosis and necrosis test (ao/eb)

The cells were plated and treated with extract as described in section 2.3. RPMI alone was used as a negative control, DMSO diluted in medium was used as the vehicle control, and doxorubicin (100 μg/mL) was used as the positive control. We followed the protocol described by [38] with adaptations. Briefly, at 24 and 48 h post-exposure, the cells were trypsinized and transferred to a Falcon tube. For analysis, acridine orange (100 μg/mL) and ethidium bromide (100 μg/mL) were mixed at a ratio of 1:1, and 2 μL of the mixture was combined with 20 μL of the cell solution on a clean slide. The sample was covered with a coverslip and analyzed under florescence microscopy (Nikon H550S). The test was performed in duplicate and 300 cells were analyzed per sample. The percentage of viable, necrosis and apoptosis cells was calculated from the formula %Cells = (number of cells of interest/total cells analyzed) *100.

### 2.10 Statistical analysis

Statistical analysis was performed using the Bioestat 5.0 software, with p≤0.05 considered statistically significant for all analyzed parameters. Normality was validated using the Kolmogorov-Smirnov test, and multiple comparisons were performed using the Tukey test followed by ANOVA [39].

## 3. Results

### 3.1 Chemical characterization of samples

The four juca extracts were chemically characterized, as presented in Table 1. We found the following: the AE extract contained phenolic compounds and carbohydrates; the ACO extract contained lipids and had a predominance of organic acids; the CLO extract contained organic acids and had a predominance of lipids; and the HEX extract contained some alcohols and had a predominance of lipids.

**Table 1:** Analysis of the global profile of the extracts composition of the *Libidibia ferrea* fruit (juca).

RT	SUBSTANCE	CLASS
**AQUEOUS ETHANOL EXTRACT**		
10.14	GLYCEROL 3TMS	Alcohol
18.14	D-FRUTOSE 5TMS	Carbohydrate
18.27	MIO-INOSITOL 6TMS	Carbohydrate
18.84	CHEMICAL ACID TMS	Phenolic
19.21	GLUCOPYRANOSIS 5TMS	Carbohydrate
20.31	GLICOSE 5TMS	Carbohydrate
25.15	1,2-BENZENODYCARBOXYLIC ACID	Phenolic
**ACETATE EXTRACT**		
7.71	OXALIC ACID 2TMS	Organic acid
10.14	GLYCEROL 3TMS	Alcohol
10.69	BUTANEDIOIC ACID 2TMS	Organic acid
10.87	PYROTARTARIC ACID 2TMS	Organic acid
12.06	PENTANOIC ACID 2TMS	Lipid
13.57	MALIC ACID 3TMS	Organic acid
14.86	PENTANODIOIC ACID 3TMS	Lipid
15.66	ARABINOIC ACID 3TMS	Organic acid
16.43	OCTANEDIOIC ACID 2TMS	Lipid
17.74	AZELAIC ACID 2TMS	Organic acid
18.12	D-GALACTOPIRANOSIL	Carbohydrate
18.29	MIO-INOSITOL 6TMS	Carbohydrate
18.86	CHEMICAL ACID 5TMS	Phenolic
19.23	GLICOSE 5TMS	Carbohydrate
19.84	BENZOIC ACID 4TMS	Phenolic
20.31	ALPHA-D-GLYCOPYRANOSIS	Carbohydrate
20.6	PALMITIC ACID TMS	Lipid
22.56	STEARIC ACID	Lipid
23.96	ACID 2-BROMOSBACICO 2TMS	Lipid-halogenated
25.12	1,2-BENZENODYCARBOXYLIC ACID	Phenolic
27.24	TETRACOSANOIC ACID TMS	Lipid
28.13	CHEMICAL ACID 5TMS	Phenolic
30.74	NO IDENTIFIED	-----
32.19	NO IDENTIFIED	-----
**CHLOROFORM EXTRACT**		
5.3	N-VALERIC ACID TMS	Lipid
6.56	ALPHA-HYDROXY ACID ISOBUTIRICACY 2TMS	Lipid
6.66	ACIDO CAPROICO TMS	Lipid
7.71	OXALIC ACID 2TMS	Organic acid
8.21	HEPTANOIC ACID TMS	Lipid
9.8	OCTANOIC ACID TMS	Lipid
10.14	GLYCEROL 3TMS	Alcohol
10.56	MALEIC ACID 2TMS	Lipid
10.67	PYROTARTARIC ACID 2TMS	Organic acid
11.36	PELARGONIC ACID TMS	Organic acid
12.93	NO IDENTIFIED	-----
13.57	MALIC ACID 3TMS	-----
14.54	TETRADECANOIC ACID TMS	Lipid
15.11	PIMELIC ACID 2TMS	Organic acid
16.41	SUBERICO ACID 2TMS	Organic acid
17.7	AZELAIC ACID 2TMS	Organic acid
18.3	MIRISTIC ACID TMS	Lipid
19.22	D-MANOSE 5TMS	Carbohydrate
19.51	N-PENTADECANOIC ACID TMS	Lipid
20.63	PALMITIC ACID TMS	Lipid
21.62	NO IDENTIFIED	-----
22.37	TES CHOLESTEROL	Steroid
22.55	STEAM ACID TMS	Lipid
23.96	ACID 2-BROMOSBACICO 2TMS	Lipid-halogenated
25.12	1,2-BENZENODYCARBOXYLIC ACID	Phenolic
25.53	MONOPALMITINE 2TMS	Lipid
25.82	DOCOSANOIC ACID TMS	Lipid
27.24	TETRACOSANOIC ACID TMS	Lipid
29.9	NO IDENTIFIED	-----
30.74	NO IDENTIFIED	-----
32.19	N-VALERIC ACID TMS	-----
**HEXANE EXTRACT**		
9.77	GLYCEROL 3TMS	Alcohol
14.14	N-DODECANOL 1TMS	Alcohol
17.89	MIRISTIC ACID 1TMS	Lipid
18.82	METILA PALMITATO	Lipid
20.26	PALMITIC ACID 1TMS	Lipid
20.75	METILA OIL	Lipid
21.03	METILA ESTEARATE	Lipid
22.2	STEAM ACID 1TMS	Lipid
22.91	METILA ARAQUIDOATE	Lipid
23.61	NO IDENTIFIED	-----
23.89	ARAQUID ACID 1TMS	Lipid
24.46	NO IDENTIFIED	-----
24.58	METILA BEENOATE	-----
26.09	METILA LIGNOCERATE	Lipid
26.57	NO IDENTIFIED	-----
26.86	TETRACOSANOIC ACID 1TMS	Lipid
27.27	NONACOSAN	Lipid
28.86	OCTACOSANOL 1TMS	Alcohol
29.7	CAMPESTEROL 1TMS	Steroid

RT: Retention time (min)

### 3.2 Antioxidant evaluation

#### 3.2.1 DPPH test

**Fig 1:**
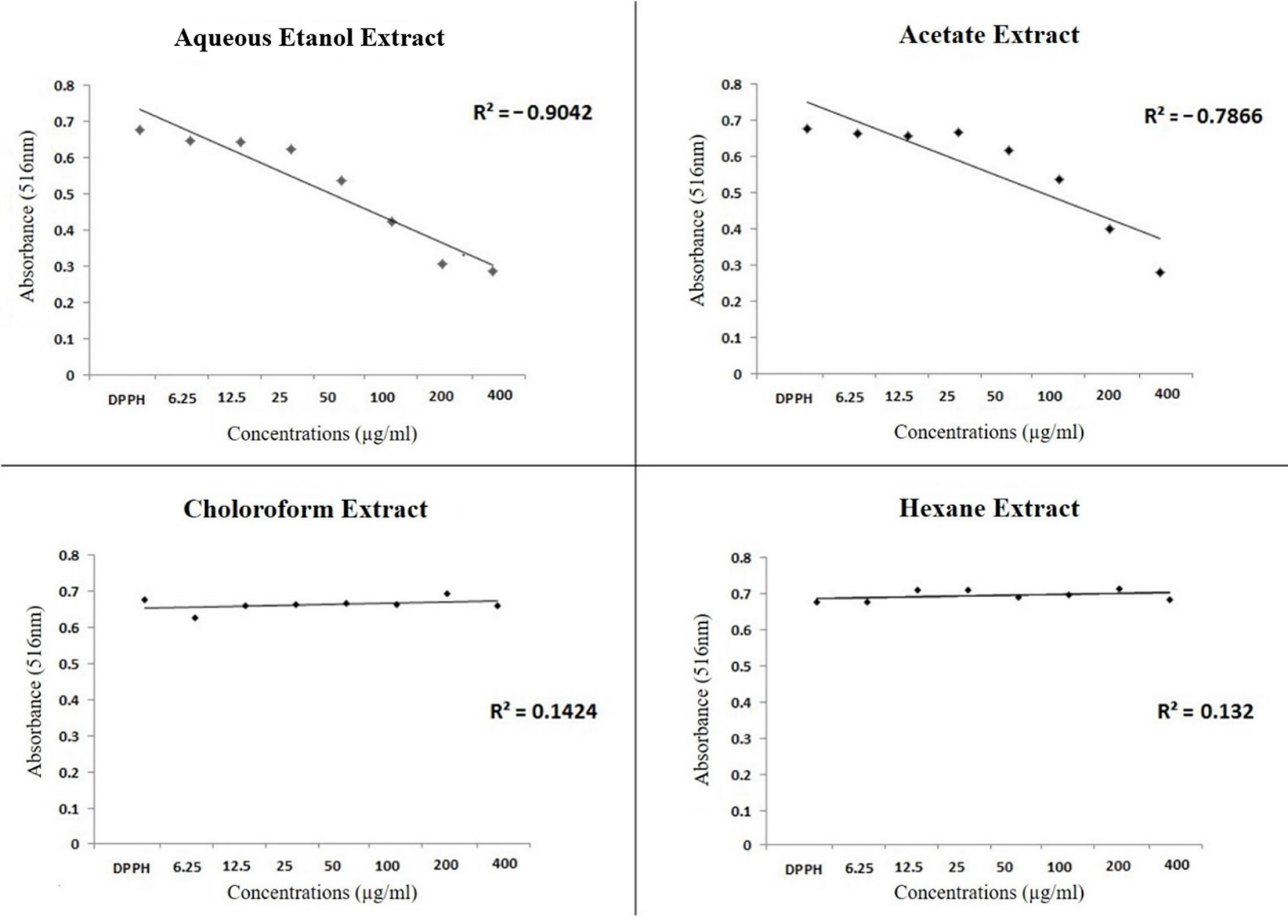
Correlation between the absorbance values according with the concentrations of the different extracts after reaction with the DPPH reagent. Linear regression test.

The DPPH free radical-capture test showed that the AE and ACO extracts exhibited dose-dependent antioxidant activity, whereas the CLO and HEX extracts showed no such activity, even at the highest tested concentration (Fig 1). The HA extract showed the highest antioxidant potential among the tested extracts, with an EC50 of 74.36 μg/mL (Table 2).

**Table 2:** Effective concentration capable of capturing 50% (EC_50_) of free radicals DPPH and ABTS after exposure to four different juca extracts.

Extract	EC_50_ (μg/mL)	
	DPPH	ABTS
**Aqueous etanol extract**	74.36	9,76
**Acetate extract**	116.10	29,13
**Chloroform extract**	>400*	>60*
**Hexane extract**	>400*	>60*

* There was no inhibition until the maximum concentration tested.

#### 3.2.2 ABTS test

The results obtained in the ABTS test corroborate those observed in the DPPH test. The EC50 of the four juca extracts are expressed in Table 2 and show the highest antioxidant activity of the AE extract with EC50 of only 9.76 μg/mL.

#### 3.2.3 ORAC test

The ORAC test confirmed the antioxidant capacity of the juca AE extract with an average of 314.29 μmol Trolox Equivalent / 100 g of extract. Thus, due to the results obtained in antioxidant assays, we focus on AE extract for the following experiments.

### 3.3 *In vitro* evaluation

**Fig 2:**
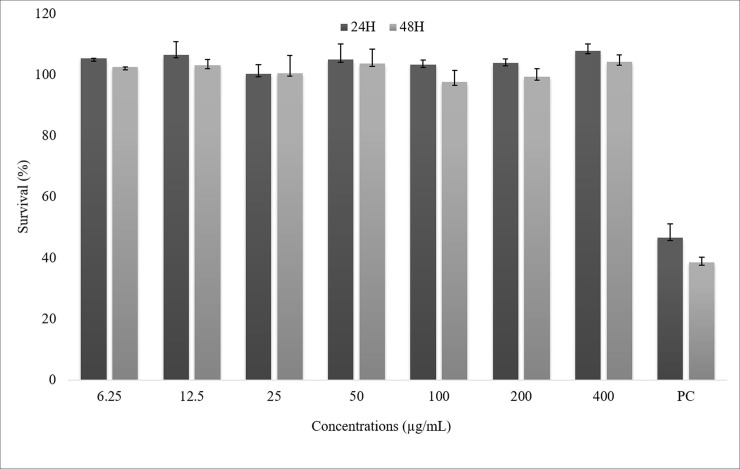
Percentage of average of survival ACP02 cells by exposure to the aqueous ethanol extract of Libidibia ferrea at 24 and 48 hours. ANOVA parametric test; Tukey-Kramer Multiple comparisons (p <0.05). In wich NC means negative control and PC means positive control.

On the cytotoxicity evaluation, the MTT assay demonstrated that the AE extract did not alter cell survival at any tested concentration (Fig 2). The Ao/Eb also did not reveal any significant increase of apoptotic or necrotic cells among the juca AE extract-treated cultures. At 24 hours, all treatment groups differed from the positive control in terms of both necrotic and apoptotic cells. At 48 hours, the treatment groups differed from the positive control group only in terms apoptosis (Table 4).

**Fig 3:**
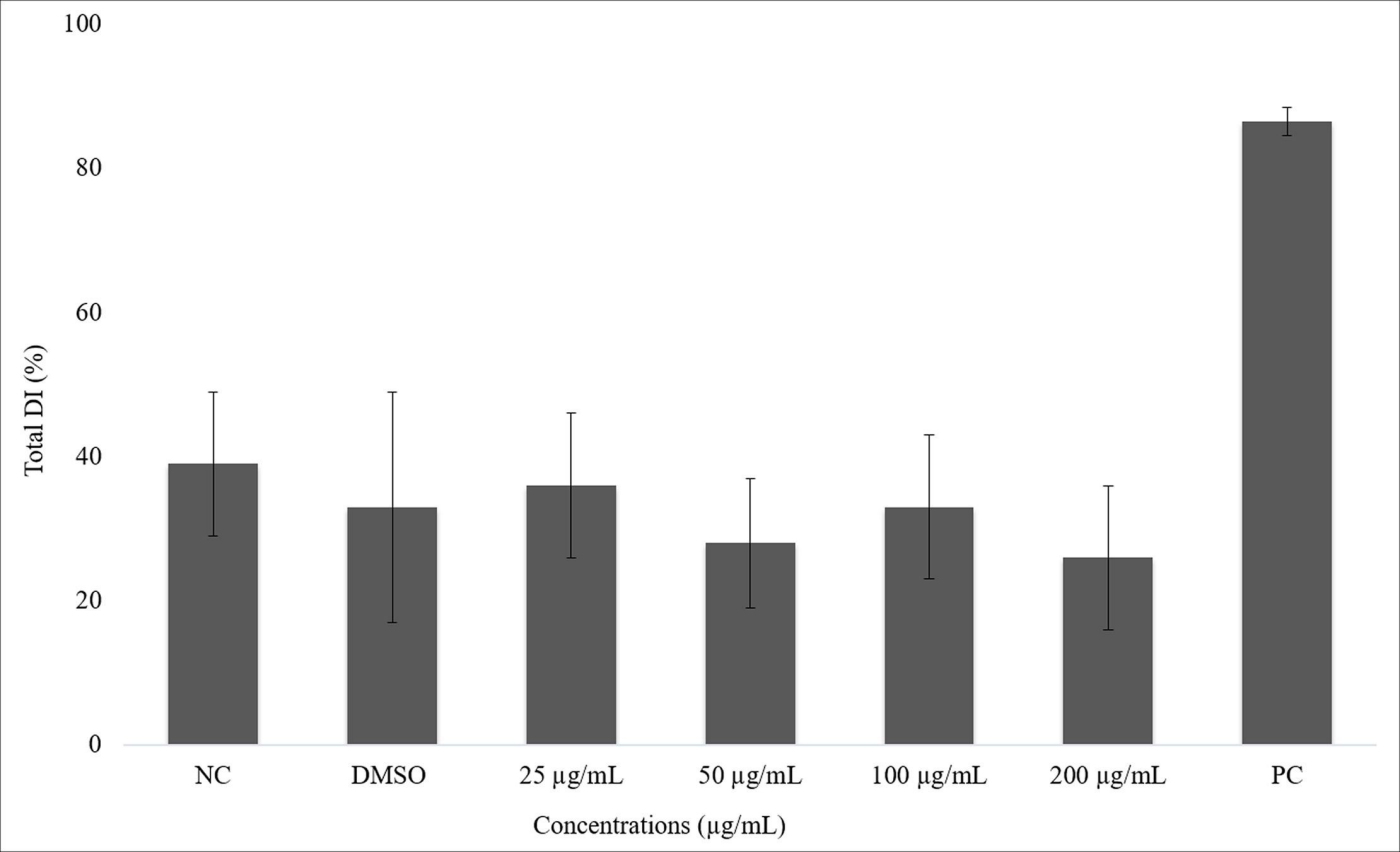
Percentage of average of total damage index in ACP02 cells after 23 hours of exposure to juca aqueous ethanol extract and it controls. ANOVA parametric test; Tukey-Kramer Multiple comparisons (p <0.05); NC means negative control, PC means positive control and * Differs from other treatments.

On the genotoxicity evaluation, the micronucleus test reveals that CBPI values did not differ for any of the treatments tested. Regarding the frequency of micronuclei, only the positive control (colchicine-treated) group differed from the other groups (Table 3). The comet assay showed no statistical difference among the AE extract-treated and negative control groups. There was an increase in the damage index of cells treated with H_2_O_2_ (positive control group) (Fig 3).

**Table 3:** Average and standard deviation of CBPI (cytokinesis blocking proliferation index) and micronucleus frequency in ACP02 cells after 24 hours exposure to juca aqueous ethanol extract and its controls.

Treatment	CBPI	MNf
**NC**	1.4(±0.09)	0.011(±0.006)
**DMSO (0.1%)**	1.3(±0.13)	0.005(±0.002)
**25 μg/ml**	1.4(±0.09)	0.005(±0.003)
**50 μg/ml**	1.3(±0.14)	0.006(±0.001)
**100 μg/ml**	1.2(±0.16)	0.005(±0.002)
**200 μg/ml**	1.4(±0.02)	0.003(±0.004)
**Colchicine (0.02 μg/ml)**	1.3(±0.1)	0.04(±0.04)*

ANOVA parametric test; Tukey-Kramer Multiple comparisons (p <0.05); NC means negative control and

* Differs from other treatments.

**Table 4:** Average and Standard Deviation (%) of viable, apoptosis and necrosis ACP02 cells at 24 and 48 hours after treatment exposure.

	24 Hour			48 Hour		
Treatments	%Viable	%Apoptosis	%Necrosis	%Viable	%Apoptosis	%Necrosis
**NC**	85 (±15)	6 (±7)^a^	9 (±8)^a^	98 (±2)	0 (±0)^a^	2 (±2)
**DMSO (0,1%)**	80 (±16)	10 (±6)^a^	10 (±10)^a^	96 (±3)	0.3 (0.5)^a^	4 (±4)
**PC**	8 (±5)	50 (±5)	8 (±10)	31 (±39)	56 (±33)	13 (±5)
**25μg/ml**	77(±18)	10 (±9)^a^	15 (±8)	67 (±2)	13 (±2)	6 (±4)
**50 μg/ml**	73 (±9)	27 (±9)	0 (±0)^a^	86 (±6)	6 (±3)	8 (±3)
**100 μg/ml**	90 (±2)	3 (±1)^a^	7 (±3)^a^	81 (±10)	2 (±0.2)^a^	17 (±11)
**200 μg/ml**	93 (±2)	2 (±0.1)^a^^,b^	5 (±3)^a^	93(±0.7)	1 (±0)^a^	2 (±0.7)

ANOVA parametric test; Tukey-Kramer Multiple comparisons (p <0.05); NC means negative control. PC means positive control with doxorubicin.

^a^Differs from other treatments.

**Fig 4:**
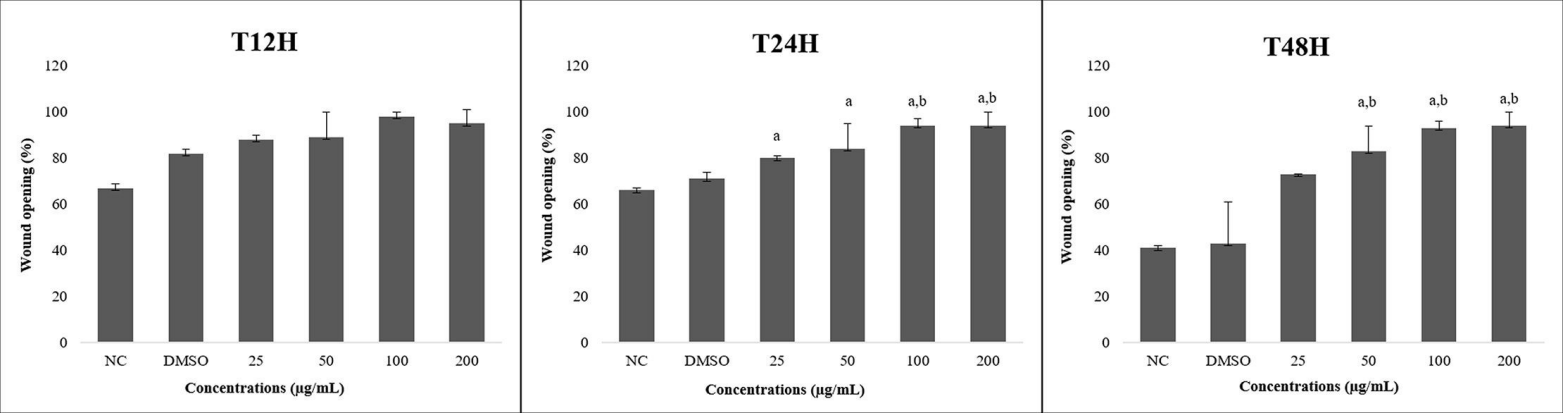
Percentage of wound opening made for the wound healing test after exposure to treatments and their respective control at times of 12, 24 and 48 hours using the ACP02 cell line. ANOVA parametric test; Tukey-Kramer Multiple comparisons (p <0.05); NC means negative control. ^a^Differs from NC. ^b^Differs from DMSO.

**Fig 5:**
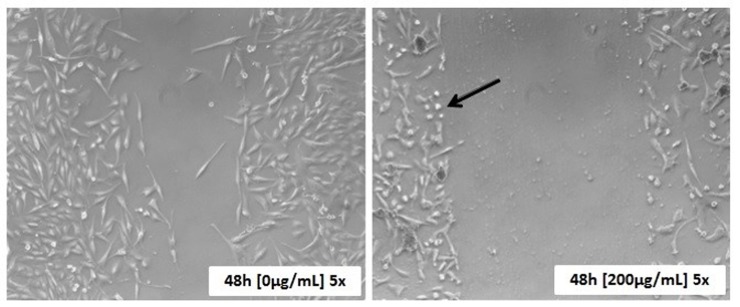
Human gastric adenocarcinoma cells (ACP02) untreated and treated with juca aqueous ethanol extract after 48 hours. **48h: 48 hours after the wound is made. 0 μg / mL and 200 μg / mL: concentrations of the juca extract. 5x: 5-fold increase**.

The wound-healing test showed that there was no significant between-group difference at 12 hours post-exposure. However, at 24 and 48 hours, ACP02 cells treated with juca AE extract exhibited decreased migratory activity, with a dose-response effect that increased at concentrations of 50 μg/mL and beyond (Fig 4). We also observed a change in the morphology of AE extract-treated cells, which exhibited decreased sizes at 24- and 48-hours post-exposure (Fig 5).

## 4. Discussion

When studying phytochemicals for their biotechnological value, it can be useful to find a product of natural origin that has an antioxidant effect. Here, we report that AE and ACO extracts of jucá exhibit antioxidant activity. Consistent with this, both extracts contained phenolic compounds, such as quinic acid, 1,2-benzenedicarboxylic acid and benzoic acid, which have been shown to block and capture free radicals [40,41]. The juca AE extract exhibited higher antioxidant activity, which was demonstrated by the three antioxidants assays performed and was therefore used for the subsequent experiments. However, we do not necessarily refer to anticarcinogenic activity. We only consider that if reactive oxygen species formation is related to various diseases (including cancer), then the ability to sequester free radicals may prevent DNA damage. For this reason, the extract with the highest antioxidant activity was chosen for toxicity and migration assays. Furthermore research can be done evaluating the effects of the other extracts.

The AE extract did not appear to have any cytotoxic, genotoxic or mutagenic potential in ACP02 cells. It did, however, appear to inhibit cell migration in this cancer cell line. [42] noted that analyses of *in vitro* migration provide good evidence for similar effects on *in vivo* migration ability, which is critical to metastasis. Thus, our results indicate that the juca AE extract may have promise as an antimetastatic agent.

Next, we examined some possible explanations for the apparent inhibition of migration in juca AE-treated ACP02 cells. Ao/Eb staining showed that this treatment did not induce apoptosis, ruling out the possibility that the observed inhibition of migration could reflect increased cell death. In vertebrates, cell movement can reflect reorganization of the cytoskeleton [43,44], which is a fiber- and protein tubule-based structure responsible for maintaining the cell shape and assisting with cell movement [45]. Indeed, we observed that the cell size was decreased in ACP02 cells treated with juca AE extract for 24 or 48 hours. We thus speculate that the extract could trigger cytoskeletal disorganization, and thereby inhibit cell migration. A similar situation was observed in a study analyzing the anti-metastatic potential of biflorin [16].

Notably, the extracellular microenvironment has been shown to modulate the organization and expression of the filaments that form the cytoskeleton [43]. The juca AE extract was found to contain myo-inositol, which is involved in cytoskeletal assembly/disassembly and the cell adhesion that is important for cell locomotion [46]. We therefore speculate that the addition of other compounds, constituent of juca AE extract, such as myo-inositol (unrelated to its antioxidant potential) to the extracellular medium can alter the organization and/or polymerization/depolymerization of the cytoskeleton, and thus prevent cell migration.

The ability of juca AE extract to inhibit cell migration suggest that this natural product might help prevent the formation of secondary tumors in an organism. The absence of cytotoxicity indicates that the extract will not act to eliminate cancerous cells. However, it might be co-treated with anticancer drugs as a means to decrease cancer progression. Our present findings therefore indicate that juca AE extract is worthy of further study as a natural product that may help prevent the progression of cancer metastasis.

Together, our results show for the first time that the aqueous ethanol extract of *Libidibia ferrea* can function as an exogenous antioxidant *in vitro*, and thus could potentially act against oxidative stress-related diseases and/or strengthen the health and well-being of an organism. Our findings suggest that this extract may counter the formation of secondary tumors (metastasis), and thus might prove useful when administered alongside treatments that eliminate cancer cells. More studies aimed at elucidating the action mechanism of the *L*. *ferrea* HA extract are needed to enable the effective use of its biological activities.

## 5. Conclusion

Together, our results show for the first time that the aqueous ethanol extract of *Libidibia ferrea* can function as an exogenous antioxidant *in vitro*, and thus could potentially act against oxidative stress-related diseases and/or strengthen the health and well-being of an organism. Our findings suggest that this extract may counter the formation of secondary tumors (metastasis), and thus might prove useful when administered alongside treatments that eliminate cancer cells. More studies aimed at elucidating the action mechanism of the *L*. *ferrea* AE extract are needed to enable the effective use of its biological activities.
