# Correction: Aqueous ethanol extract of *Libidibia ferrea* (Mart. Ex Tul) L.P. Queiroz (juca) exhibits antioxidant and migration-inhibiting activity in human gastric adenocarcinoma (ACP02) cells

**DOI:** 10.1371/journal.pone.0257134

**Published:** 2021-09-01

**Authors:** The PLOS ONE Staff

An additional affiliation is missing for the third author. Karina Motta Melo is also affiliated with Universidade Federal Rural da Amazônia, Campus Tomé Açu, Tomé Açu, Pará, Brazil.

There are errors in Figs 2 and 3. The NC column is missing from Fig 2, and the asterisk above the PC column is missing from Fig 3. The authors have provided corrected versions here. The publisher apologizes for the error.

There is an error in the Fig 2 caption. Please see the complete, correct Fig 2 caption here.

**Fig 2:**
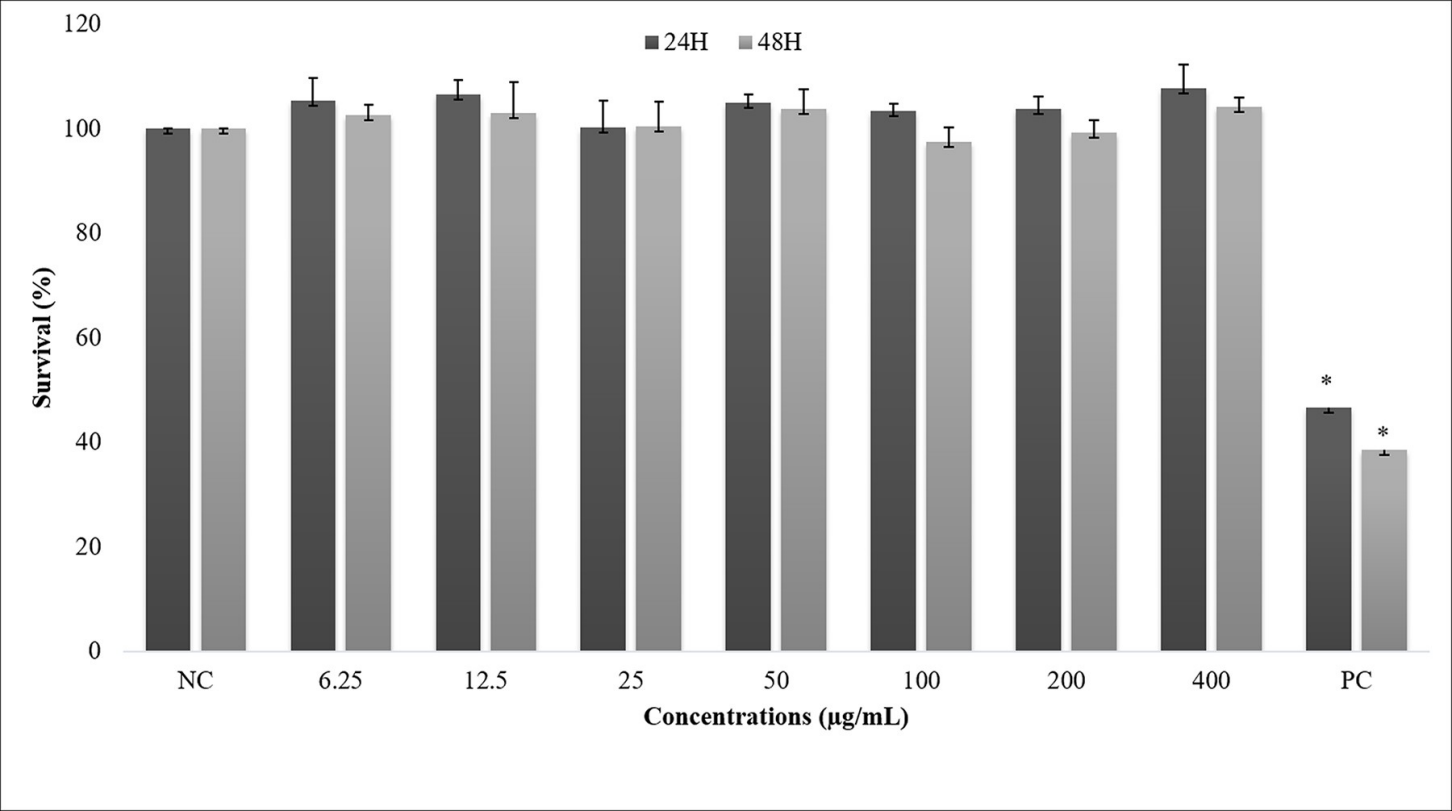
Percentage of average of survival ACP02 cells by exposure to the aqueous ethanol extract of *Libidibia ferrea* at 24 and 48 hours. ANOVA parametric test; Tukey-Kramer Multiple comparisons (p <0.05). In which NC means negative control and PC means positive control and * Differs from other treatments.

**Fig 3:**
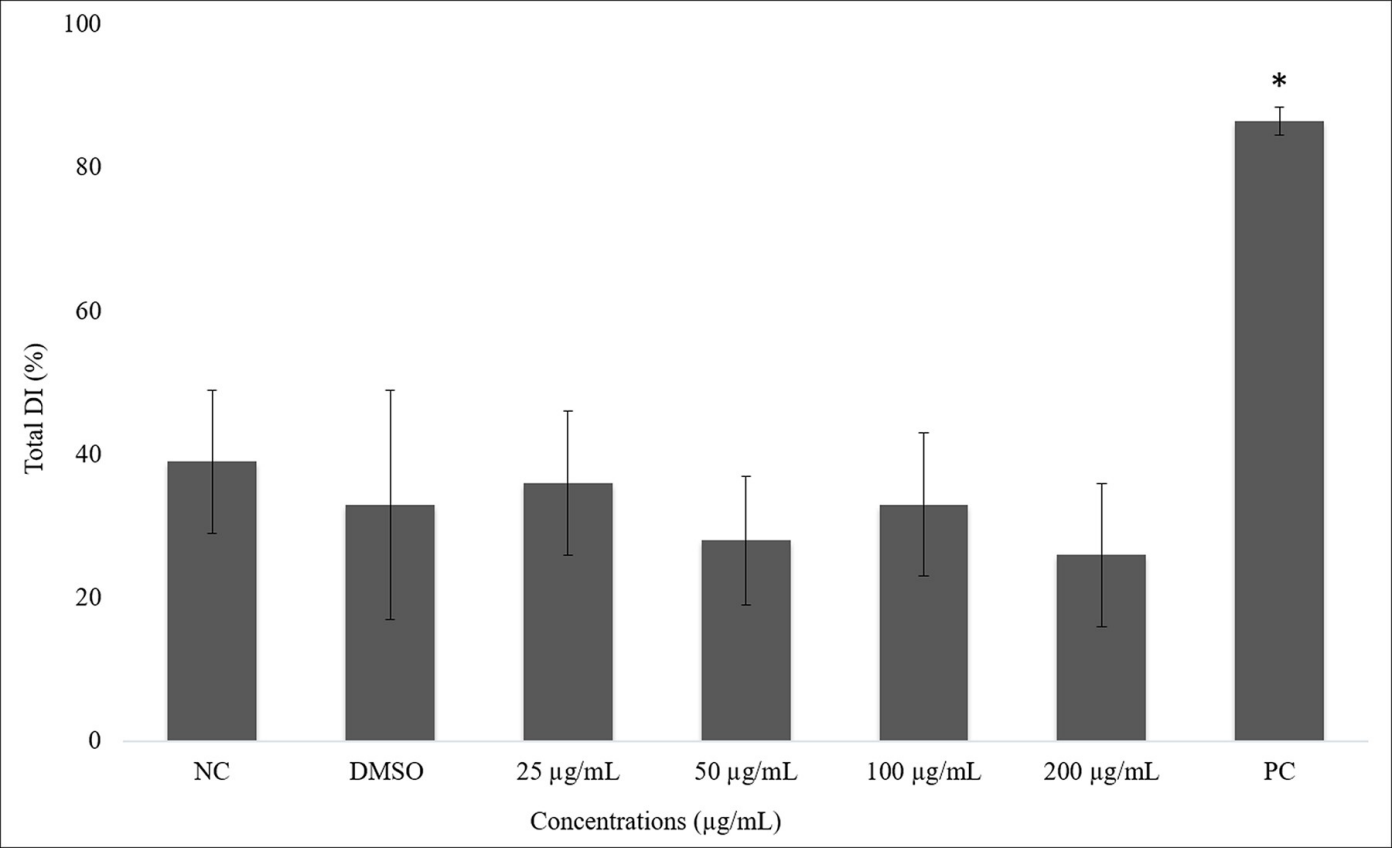
Percentage of average of total damage index in ACP02 cells after 23 hours of exposure to juca aqueous ethanol extract and it controls. ANOVA parametric test; Tukey-Kramer Multiple comparisons (p <0.05); NC means negative control, PC means positive control and * Differs from other treatments.
